# Endoscopic Anatomical Study of the Skull Base with Poor Pneumatization: A Quantitative Analysis Based on Cadaveric Specimens

**DOI:** 10.3390/brainsci16101132

**Published:** 2026-10-20

**Authors:** Benlin Wang, Qiaowei He, Nan Chi, Hongtao Zhang, Xicheng Song

**Affiliations:** 1 Department of Neurosurgery, Yantai Yuhuangding Hospital, Qingdao University, Yantai 264000, China; wbl1115ever@126.com (B.W.); joetomato@126.com (Q.H.); 13854580066@163.com (N.C.); 2 Department of Otorhinolaryngology, Head and Neck Surgery, Yantai Yuhuangding Hospital, Qingdao University, Yantai 264000, China; 3 Shandong Provincial Key Laboratory of Neuroimmune Interaction and Regulation, Yantai Yuhuangding Hospital, Yantai 264000, China; 4 Shandong Provincial Clinical Research Center for Otorhinolaryngologic Diseases, Yantai Yuhuangding Hospital, Yantai 264000, China; 5 Yantai Key Laboratory of Otorhinolaryngologic Diseases, Yantai Yuhuangding Hospital, Qingdao University, Yantai 264000, China

**Keywords:** poor pneumatization, endoscopic anatomy, skull base tumor, quantitative analysis

## Abstract

**Highlights:**

**What are the main findings?**

- In poorly pneumatized specimens, classical sphenoid landmarks (sellar floor, ICA prominence, clival recess) are absent, whereas the vidian canal-foramen rotundum complex is identifiable in 100% of sides.
- Quantitative data show gradual clival widening, relative symmetry of occipital condyles, and a mean petrous apex drilling volume of approximately 1.0 cm^3^ (45–48% of total), with a maximum of 63.8% in one specimen.

**What are the implications of the main findings?**

- When pneumatization is poor, surgeons should rely on constant osseous coordinates (vidian canal, supracondylar groove) rather than air-cell-dependent prominences for safe localization.
- The measured morphometric data provide anatomical reference values; however, they should not be regarded as validated safety thresholds, and clinical validation is required.

**Abstract:**

Background: The endoscopic endonasal approach (EEA) has been widely applied in the surgical treatment of skull base tumors. However, poor pneumatization of the skull base—such as inadequate sphenoid sinus pneumatization and dense clival bone—can severely obscure key anatomical landmarks, significantly increasing surgical risks. Systematic quantitative studies on endoscopic anatomical landmarks under conditions of poor pneumatization remain limited to date. Methods: Using 10 lightly embalmed adult cadaveric head specimens with red-blue silicone injection, we performed a stepwise dissection of the nasal cavity; sphenoid sinus; cavernous sinus (CS); upper, middle, and lower clivus; craniovertebral junction (CVJ); and lateral skull base regions via the expanded endoscopic EEA with a 0° rigid endoscope. Particular emphasis was placed on observing variations in the landmarks within the sphenoid sinus under conditions of poor pneumatization, and morphometric measurements were obtained for key structures, including the clivus and its adjacent areas, occipital condyles (OC), hypoglossal canal (HC), petrous apex (PA), and vidian canal (VC). Results: In poorly pneumatized specimens, the clival recess (CR) within the sphenoid sinus is often not observable, and the optic nerve prominence and the internal carotid artery (ICA) prominence are indistinct. Quantitative data showed that the total length of the clivus was 28.7 ± 2.1 mm, with gradual widening from superior to inferior (upper clivus: 20.8 ± 2.0 mm; middle clivus: 22.7 ± 2.8 mm; lower clivus: 29.2 ± 3.9 mm). The average drillable area of the entire clivus was 8.6 ± 1.1 cm^2^, and the PA could be safely drilled by approximately 45–48% (about 1.0 cm^3^) via the EEA. The length of the VC ranged from 17.1 to 17.8 mm, and its distance from the foramen rotundum (FR) was approximately 7.8–8.0 mm, which may serve as a useful anatomical reference for localizing the horizontal petrous segment of the ICA. Conclusions: In cases of poor pneumatization of the skull base, the osseous and vascular prominences are often obscured under EEA, necessitating coordinate-based localization using quantitative anatomical data. The present study provides segmental clival widths, three-dimensional measurements of the OC-HC complex, the VC-FR interval, and the safe drillable volume of the PA, thereby furnishing a quantitative reference for precise and safe surgery in the poorly pneumatized skull base.

## 1. Introduction

The endoscopic endonasal approach (EEA), with its advantages of direct access to the ventral skull base and avoidance of brain retraction, has become an important surgical modality for various tumors involving the clival region, sellar area, cavernous sinus, and lateral skull base [1,2,3,4]. However, the successful implementation of EEA is highly dependent on the accurate intraoperative identification of osseous landmarks and neurovascular prominences within the sphenoid sinus, clivus, and adjacent regions.

Sphenoid sinus pneumatization is a progressive process that begins in early childhood (around 3 years of age) and is largely completed by adolescence (approximately 16 years of age), with significant individual variability in the degree of pneumatization [5]. Hammer and Radberg first introduced a four-tiered classification scheme for sphenoid sinus pneumatization, stratifying types according to their extent relative to the sella turcica. Following this foundational work, a considerable body of literature has documented substantial variability in the reported incidences of these subtypes among different patient cohorts [6,7,8]. In the presence of these anatomical variants, the opticcarotid recess (OCR), the carotid artery prominence, and the clival recess (CR), which are ordinarily clearly identifiable in well-pneumatized sphenoid sinuses, are often absent or poorly defined. Further studies by Kang et al. [9] have confirmed that when sphenoid sinus pneumatization is poor, the internal carotid artery (ICA) prominence is not obvious, and the intersphenoid septum is often attached to or closely adjacent to the paraclival ICA. In addition, poor pneumatization of the lateral recess of the sphenoid sinus (LRSS) has also been shown to increase surgical difficulty and the risk of neurovascular injury [10].

Prior anatomical investigations have largely been conducted on well-pneumatized specimens or imaging datasets [11,12,13], and there remains a paucity of systematic quantitative analyses of endoscopic anatomical landmarks specifically under conditions of poor sphenoid sinus pneumatization. To fill this gap, the present study was designed with the following objectives: (1) to replicate the critical steps of the expanded EEA in human cadaveric heads under a simulated scenario of poor pneumatization; (2) to perform precise morphometric quantification of the clivus and its surrounding neurovascular structures; and (3) to establish a surgical localization strategy based on rigid osseous coordinates rather than on pneumatization-dependent osseous bulges, thereby furnishing fundamental anatomical baseline references for the clinical management of various skull base pathologies.

## 2. Materials and Methods

### 2.1. Specimens and Equipment

The 10 specimens were lightly embalmed with a standard solution (e.g., 10% formalin) via arterial perfusion, followed by injection of red- and blue-colored silicone into the arterial and venous systems, respectively, to facilitate identification of vascular structures. The 10 adult cadaveric head specimens (20 sides) were obtained from a legitimate body donation program. Because the specimens were fully de-identified prior to dissection, complete demographic data were not available. Poor pneumatization was defined as a sphenoid sinus cavity that did not extend beyond the level of the tuberculum sellae, with absence of the sellar floor and clival recess prominences under endoscopic view. Based on this criterion, all 20 sides were classified as poorly pneumatized; no well-pneumatized control sides were available in this specimen set.

A Karl Storz 0° rigid endoscope (4 mm in diameter, 18 cm in length) was connected to a light source and a video recording system, a high-speed drill (3–4 mm diamond burr), and the microsurgical instruments used for dissection. The specimens were placed in the supine position, and the head was fixed in a neutral position using a Mayfield skull clamp.

### 2.2. Measurement of Linear Dimensions, Areas, and Volumes

Linear dimensions were measured with a flexible ruler and a vernier caliper. Resectable areas were calculated from the measured linear dimensions using standard geometric formulas (e.g., length × width for rectangular areas). Resectable volumes were estimated by measuring the length, width, and height of the drilled cavity with a flexible ruler and calculating the volume using the formula for an ellipsoid or a rectangular prism, as appropriate. All dissections and measurements were repeated twice by the same neurosurgeon.

### 2.3. Statistical Analysis

All statistical analyses were performed using IBM SPSS Statistics version 25.0. Descriptive statistics are presented as mean ± standard deviation (SD) for continuous variables, and as counts and percentages for categorical variables. The normality of each continuous variable was assessed using the Shapiro–Wilk test. For bilateral comparisons, paired-samples *t*-tests were used when the differences between paired observations were normally distributed; otherwise, the Wilcoxon signed-rank test was applied. Given the exploratory nature of this morphometric study and the relatively small sample size, corrections for multiple comparisons were not applied, and the significance level was set at a two-sided *p* < 0.05. All *p* values are reported as two-tailed.

## 3. Anatomical Procedures and Structural Observation

### 3.1. Transsphenoidal Approach and Sphenoid Sinus Exploration

**Figure 1:**
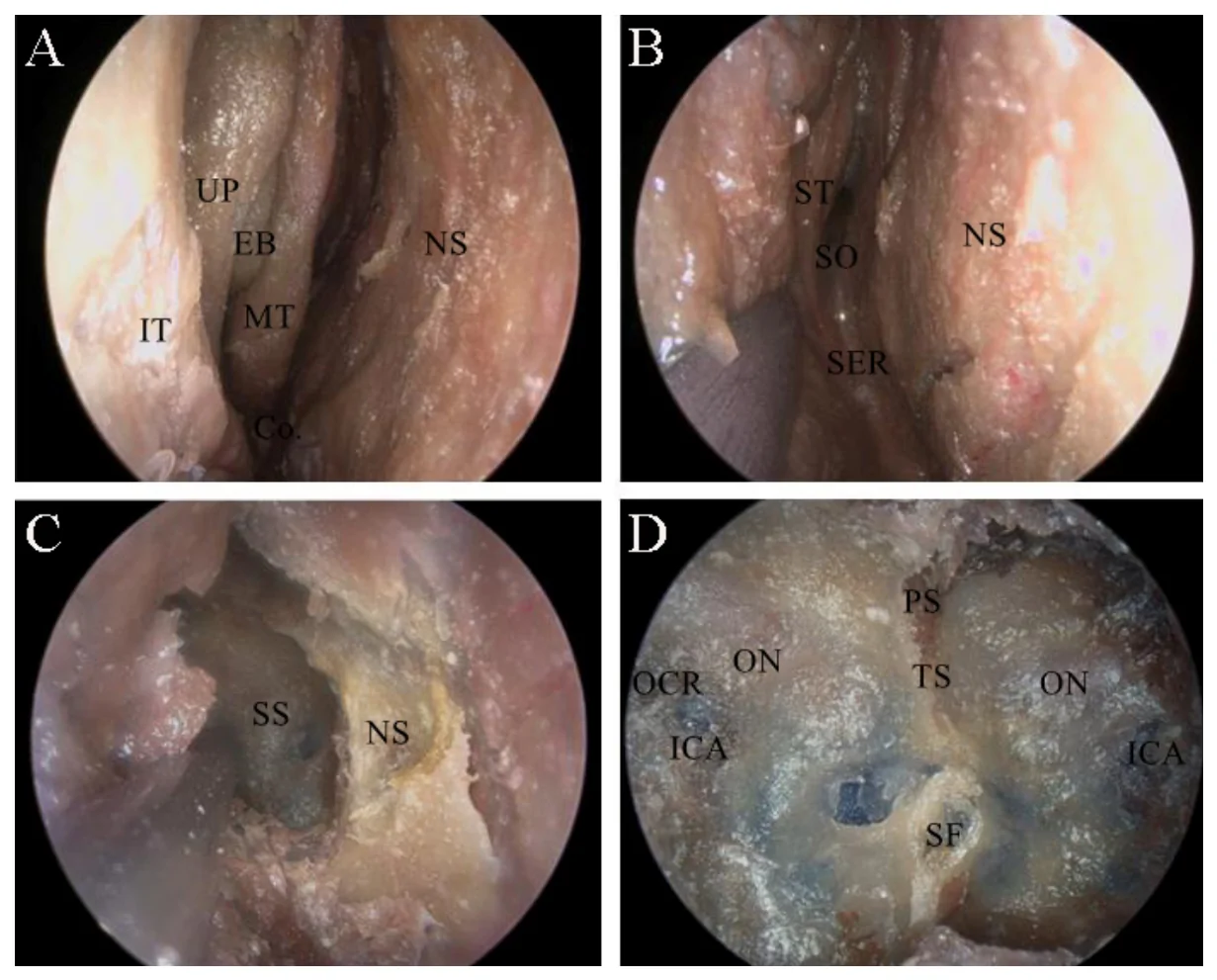
Endoscopic endonasal transsphenoidal approach. (**A**) Endoscopic view of the right nasal cavity demonstrating the major anatomical structures. (**B**) Endoscopic identification of the sphenoid sinus ostium between the superior turbinate and the nasal septum, along the posterior nasal cavity. (**C**) The posterior portion of the nasal septum was detached from the sphenoid rostrum and resected, thereby exposing the contralateral sphenoid sinus. (**D**) After removal of the intrasphenoid septations, the bilateral sphenoid sinus cavities were fully exposed, but these landmarks were only partially identifiable, and their prominences were indistinct. EB, ethmoid bulla; ICA, internal carotid artery; IT, inferior turbinate; MT, middle turbinate; NS, nasal septum; OCR, opticocarotid recess; ON, optic nerve; PS, planum sphenoidale; SER, sphenoethmoidal recess; SF, sellar floor; SO, sphenoid sinus ostium; SS, sphenoid sinus; ST, superior turbinate; TS, tuberculum sellae; UP, uncinate process.

The endoscopic procedure was performed via the right nostril, with identification of the inferior turbinate (IT), middle turbinate (MT), and nasal septum (NS) (Figure 1A). The choana and the sphenoethmoidal recess (SER) were subsequently confirmed, and the sphenoid sinus ostium (SO) was sought. Of note, in specimens with poor pneumatization, the SO was frequently covered by the superior turbinate (ST) or was otherwise difficult to locate. When the SO was not visible, blunt dissection was performed along the SER, between the ST and the NS, to penetrate the thin osseous wall and gain entry into the sphenoid sinus. After opening the anterior wall of the sphenoid sinus, the intrasinus septations were removed, with particular attention paid to how poor pneumatization affected the visualization of the sellar floor (SF), tuberculum sellae (TS), optic nerve (ON), and ICA prominences (Figure 1B–D). At this stage, the CR was generally not discernible under conditions of poor pneumatization.

### 3.2. Basic Principles of Expanded EEA and the Transplanum Approach

**Figure 2:**
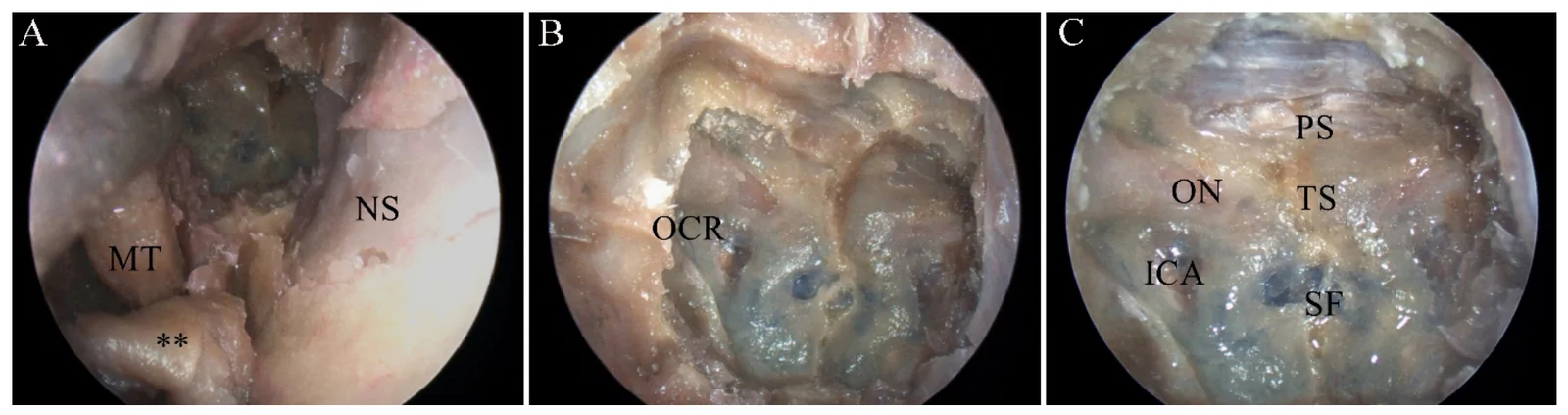
Stepwise dissection of transplanum sphenoidale approach. (**A**) Right middle turbinectomy was performed for the expanded EEA. (**B**) The anterior wall of the sphenoid sinus was circumferentially widened in a ring-like fashion, with the lateral limit extending to the OCR. (**C**) The tuberculum sellae was mobilized, and the superior half of the sellar floor along with the bony planum sphenoidale was drilled away. ICA, internal carotid artery; MT, middle turbinate; NS, nasal septum; OCR, opticocarotid recess; ON, optic nerve; PS, planum sphenoidale; SF, sellar floor; TS, tuberculum sellae; ** nasoseptal flap.

**Figure 3:**
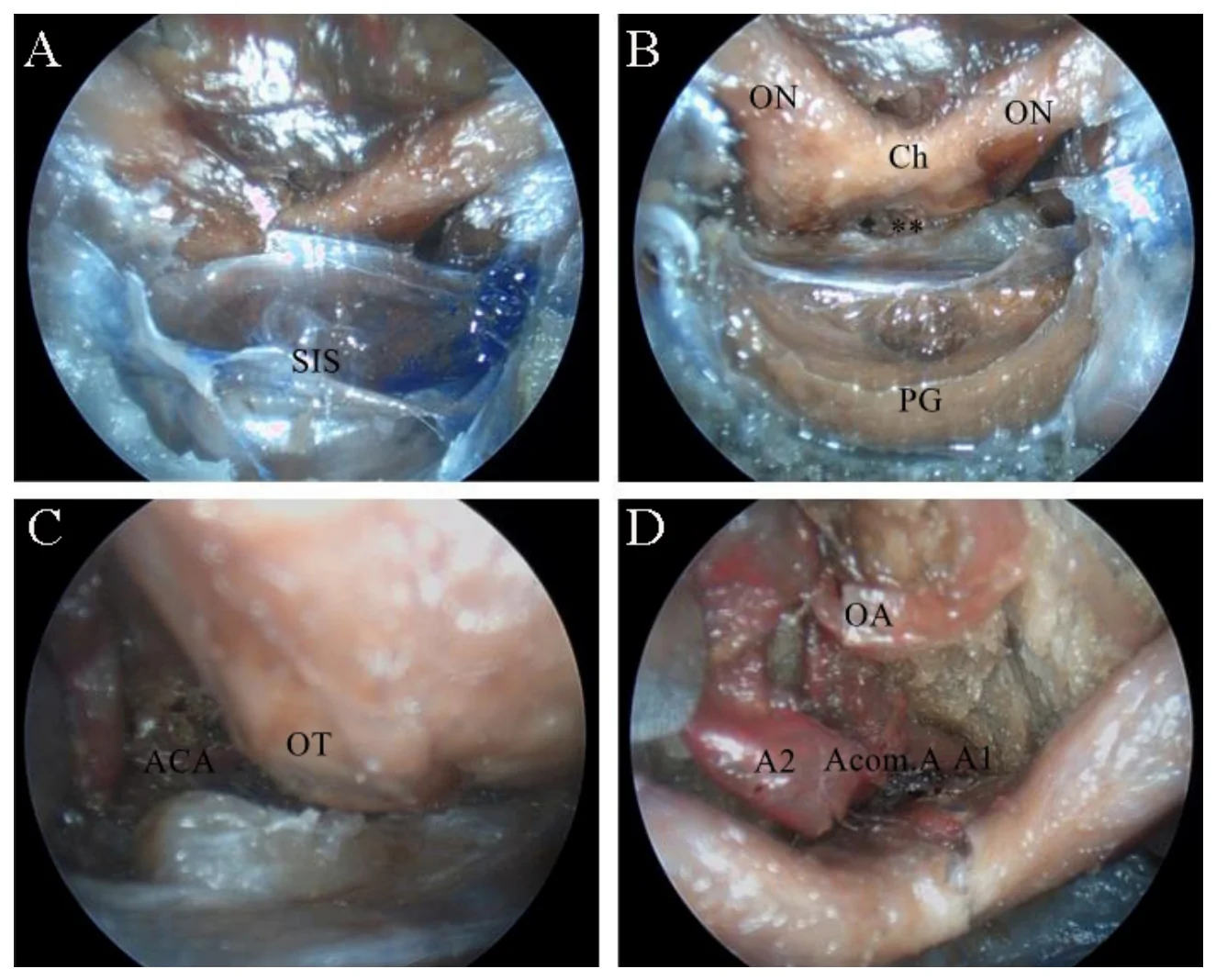
After dural opening, the superior and inferior surgical fields centered on the optic chiasm were exposed. (**A**) Following dural opening, the intradural structures were visualized. (**B**,**C**) The infrachiasmatic view was exposed. (**D**) Exposure of the suprachiasmatic view revealed the anterior portion of the circle of Willis. A1, A1 segment of the anterior cerebral artery; A2, A2 segment of the anterior cerebral artery; ACA, anterior cerebral artery; Acom.A, anterior communicating artery; Ch, optic chiasm; OA, ophthalmic artery; ON, optic nerve; OT, optic tract; PG, pituitary gland; SIS, superior intercavernous sinus; ** pituitary stalk.

To obtain bilateral working space, the right MT was resected, the left MT was lateralized, and the posterior portion of the nasal septum was removed (Figure 2A). For suprasellar extension, the ST and the posterior ethmoid cells were resected. Care was taken to preserve the posterior ethmoidal artery (PEA) and the cribriform plate to avoid olfactory injury. After adequate exposure of the sphenoid sinus cavity, the TS and the posterior part of the planum sphenoidale (PS) were drilled away, extending bilaterally to the OCR (Figure 2B,C). Following dural opening, the optic chiasm, pituitary stalk, anterior cerebral artery (ACA), and the anterior portion of the circle of Willis were visualized (Figure 3).

### 3.3. Anatomical Dissection of the Cavernous Sinus Region

**Figure 4:**
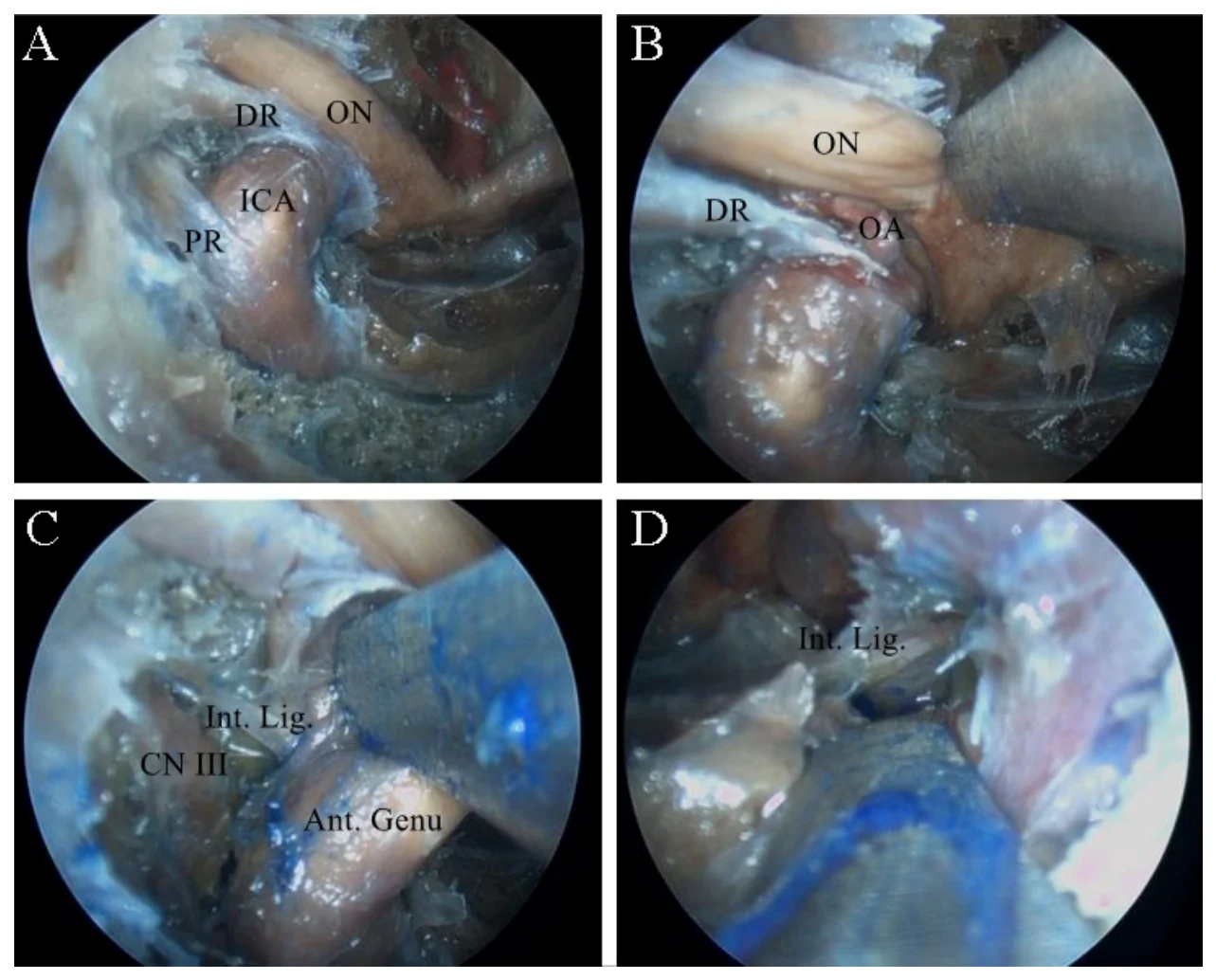
Parasellar ICA and the superior compartment of the cavernous sinus. (**A**,**B**) Exposure of the right parasellar ICA, demonstrating the cavernous and paraclinoid segments of the ICA (located between the proximal and distal dural rings), with the OA visualized inferior to the ON posterior to the distal dural ring. (**C**) The superior compartment of the right CS, showing the interclinoid ligament, the CN III, and the anterior genu of the cavernous ICA. (**D**) The interclinoid ligament in the superior compartment of the left CS. Ant. Genu, anterior genu of the cavernous ICA; CN III, oculomotor nerve; DR, distal dural ring; ICA, paraclinoid ICA; Int. Lig., interclinoid ligament; OA, ophthalmic artery; ON, optic nerve; PR, proximal dural ring.

**Figure 5:**
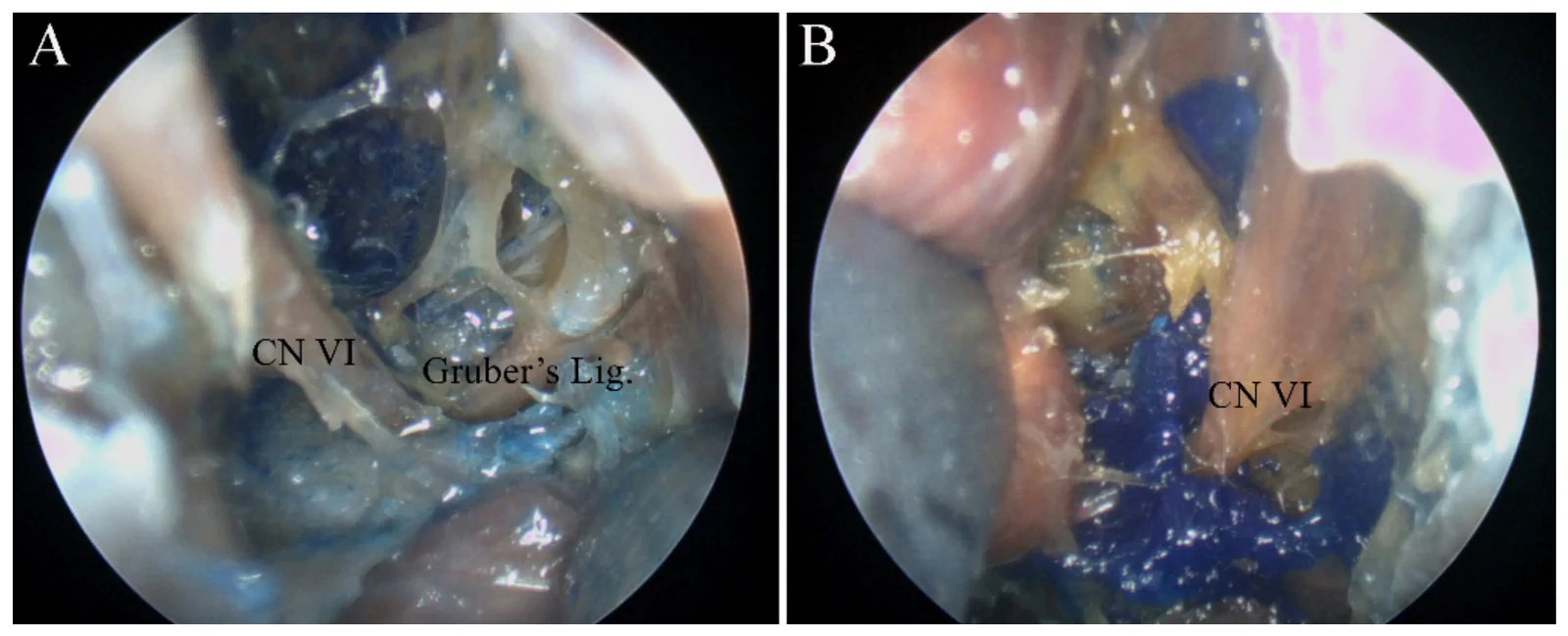
Posterior compartment of the cavernous sinus. (**A**) The posterior compartment of the right CS, located behind the short vertical segment of the cavernous ICA. The CN VI lies at the most inferior portion of this compartment and enters the CS after passing inferior to the petrosphenoid (Gruber’s) ligament. (**B**) The posterior compartment of the left CS, revealing a conspicuous venous confluence. CN VI, abducens nerve; Gruber’s Lig., Gruber’s ligament/petrosphenoid ligament.

**Figure 6:**
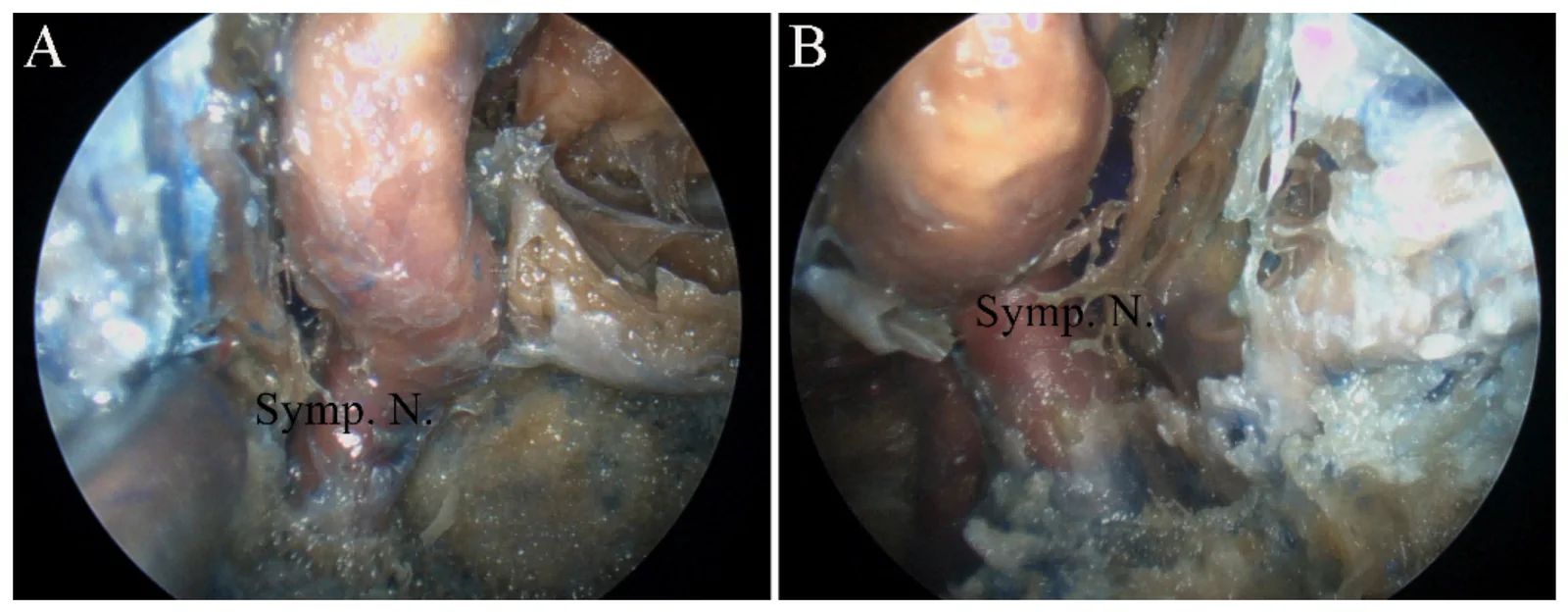
Inferior compartment of the cavernous sinus. (**A**) The inferior compartment of the right CS, situated below the horizontal segment and the anterior genu of the ICA, and anterior to the short vertical segment. Several sympathetic nerve fibers were observed intertwining on the surface of the ICA. (**B**) The inferior compartment of the left CS. Symp. N., sympathetic plexus.

**Figure 7:**
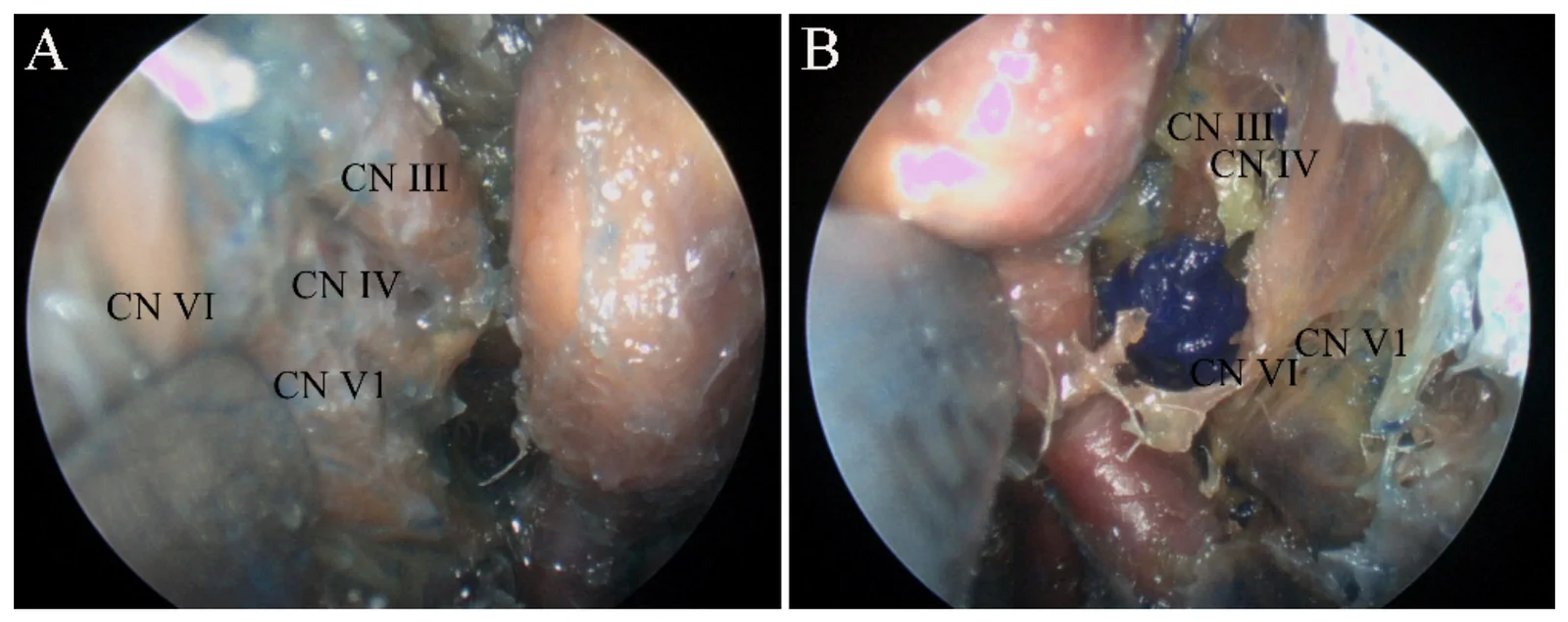
Lateral compartment of the cavernous sinus. (**A**) The lateral compartment of the right CS, located lateral to the horizontal segment and the anterior genu of the ICA, showing the cranial nerves situated within this compartment. (**B**) The lateral compartment of the left CS. CN III, oculomotor nerve; CN IV, trochlear nerve; CN V1, ophthalmic division of the trigeminal nerve; CN VI, abducens nerve.

The CS is divided into four compartments: superior, posterior, inferior, and lateral [3]. The superior compartment, located above the horizontal segment of the ICA, is distinguished by the interclinoid ligament (connecting the anterior and posterior clinoid processes) as a key landmark; medially it is bordered by the paraclinoid ICA, and laterally by the oculomotor nerve (CN III) (Figure 4). The posterior compartment, situated behind the short vertical segment of the ICA, contains the abducens nerve (CN VI), which enters the CS through Dorello’s canal, inferior to Gruber’s ligament (Figure 5). The inferior compartment, lying below the horizontal segment of the ICA, contains the sympathetic plexus surrounding the ICA (Figure 6). The lateral compartment, located lateral to the horizontal segment of the ICA, contains CN III, CN IV, and the ophthalmic division of the trigeminal nerve (V1) (Figure 7).

### 3.4. Anatomy of the Superior, Middle, and Inferior Clival Approaches

**Figure 8:**
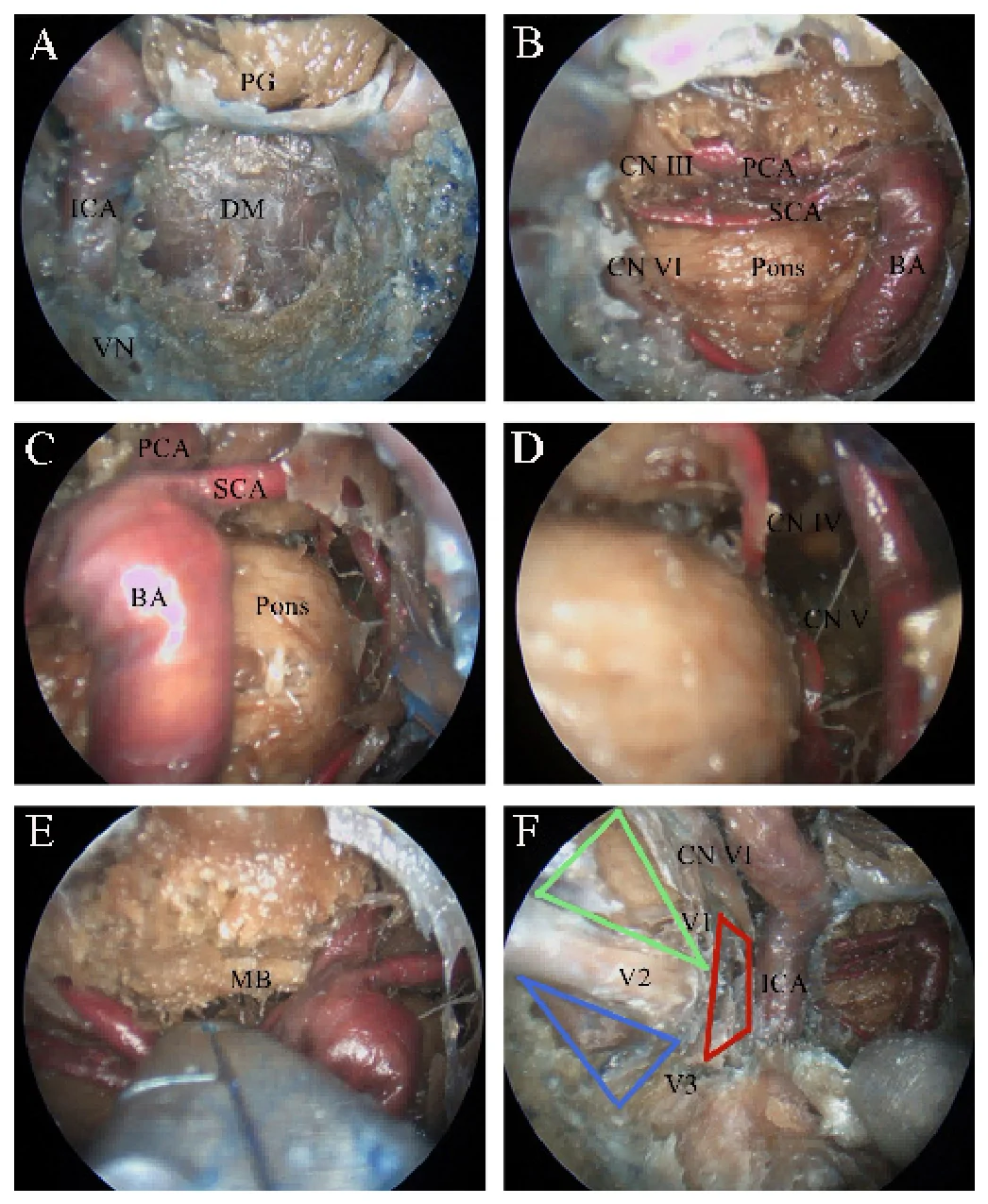
Stepwise dissection of the superior clival approach. (**A**) The bone of the sellar floor and the superior clivus was drilled away, exposing the underlying dura mater and the paraclival ICA. (**B**,**C**) After opening the subsellar and superior clival dura, the upper half of the anterior surface of the pons and the prepontine cistern were exposed, with the pontomesencephalic junction visible at the most superior aspect, along with the upper segment of the BA and its terminal branches. (**D**) With the endoscope advanced further to the left, the CN IV and the prominent trigeminal nerve root (CN V) were visualized. (**E**) Through an expanded superior clival approach, the dorsum sellae was removed and the pituitary gland was elevated, thereby exposing the interpeduncular cistern and the bilateral MB. (**F**) Lateral extension of the approach to the right side revealed two lateral CS triangles and the quadrangular space. BA, basilar artery; CN III, oculomotor nerve; CN IV, trochlear nerve; CN V, trigeminal nerve; CN VI, abducens nerve; DM, dura mater; ICA, paraclival internal carotid artery; MB, mammillary bodies; PCA, posterior cerebral artery; PG, pituitary gland; Pons, pons; SCA, superior cerebellar artery; V1, ophthalmic division of the trigeminal nerve; V2, maxillary division of the trigeminal nerve; V3, mandibular division of the trigeminal nerve; VN, vidian nerve. Color-coded annotations: green triangle, anteromedial triangle of the cavernous sinus; blue triangle, upper part of the anterolateral triangle of the cavernous sinus; red area, quadrangular space.

Superior clivus: bounded by the dural entry point of the CN VI and the upper margin of the foramen lacerum (FL). The bone from the sellar floor to the superior clivus was drilled away, exposing the dura mater and the paraclival ICA (Figure 8A). After dural opening, the upper segment of the basilar artery (BA), the posterior cerebral artery (PCA), and the superior cerebellar artery (SCA) were visualized (Figure 8B,C). Following elevation of the pituitary gland and drilling of the dorsum sellae, the mammillary bodies (MB) were exposed (Figure 8E). Lateral extension revealed the anteromedial triangle of the CS and the quadrangular space (Figure 8F).

**Figure 9:**
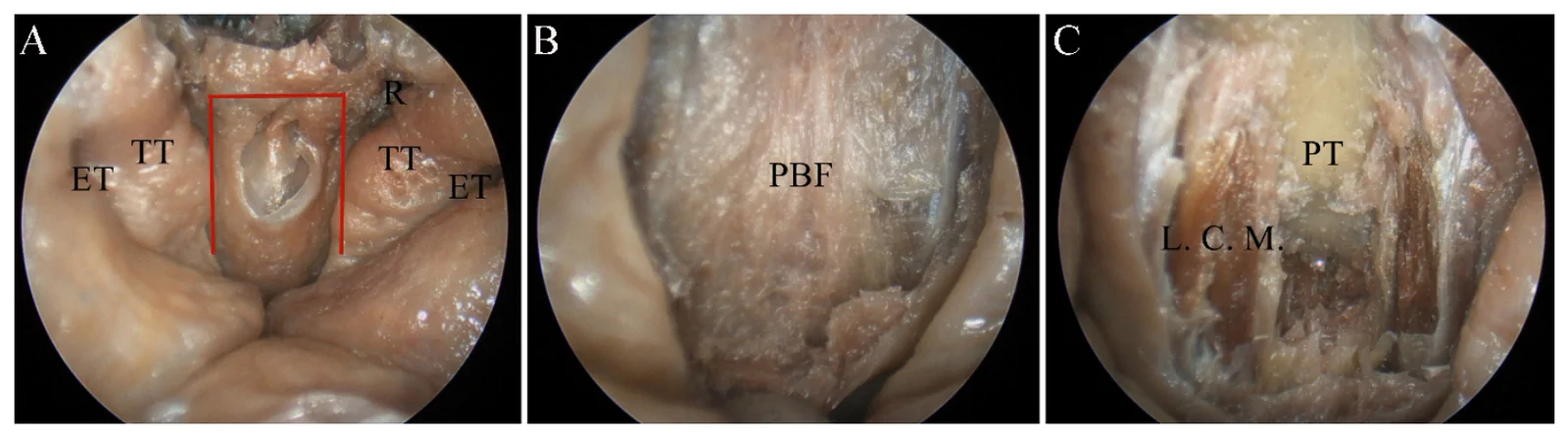
Pharyngobasilar fascia dissection. (**A**) The red line indicates the planned inverted U-shaped incision in the most anterior nasopharyngeal mucosa, which was then reflected toward the soft palate. (**B**) The overlying mucosa was removed, exposing the PBF. (**C**) The PBF was resected, revealing the underlying longus capitis muscle (LCM) and the centrally located pharyngeal tubercle (PT). ET, Eustachian tube; L.C.M., longus capitis muscle; PBF, pharyngobasilar fascia; PT, pharyngeal tubercle; R, pharyngeal recess; TT, torus tubarius.

**Figure 10:**
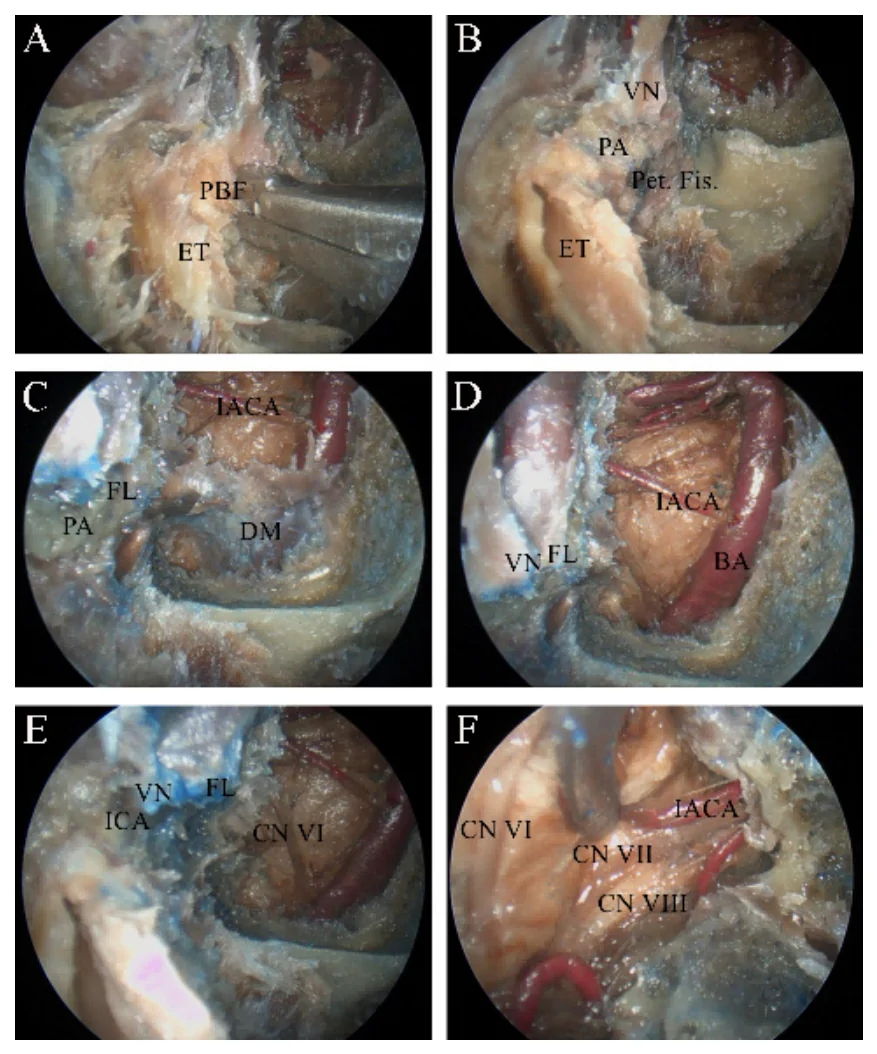
Stepwise dissection of the middle clival approach. (**A**,**B**) The fibrocartilage filling the petroclival fissure and extending to the foramen lacerum (FL) was removed on the right side, exposing the petroclival fissure and the most medial aspect of the petrous apex (PA). (**C**,**D**) The middle clival bone was drilled away, and the dura mater was opened to reveal the intradural structures, showing the origin of the right anterior inferior cerebellar artery (AICA). (**E**) After drilling of the right PA, the horizontal petrous segment of the ICA was visualized inferior to the VN, along with the foramen lacerum segment of the ICA. (**F**) Neurovascular structures within the left middle clivus, demonstrating the CN VI, the origin of the CN VII and CN VIII, their cisternal course toward the internal auditory meatus, and the premeatal segment of the AICA. BA, basilar artery; CN VI, abducens nerve; CN VII, facial nerve; CN VIII, vestibulocochlear nerve; DM, dura mater; ET, Eustachian tube; FL, foramen lacerum; AICA, anterior inferior cerebellar artery; ICA, horizontal petrous segment of the internal carotid artery; PA, petrous apex; PBF, pharyngobasilar fascia; Pet. Fis., petroclival fissure; VN, vidian nerve.

Middle clivus: bounded inferiorly by the level of the glossopharyngeal nerve canal (the superomedial margin of the jugular foramen). The pharyngobasilar fascia (PBF) was addressed (Figure 9), exposing the longus capitis muscle (LCM) and the pharyngeal tubercle (PT). The fibrocartilage of the petroclival fissure was removed, and the middle clival bone was drilled away (Figure 10A–C). After dural opening, the cisternal segment of the CN VI, the facial and vestibulocochlear nerves (CN VII and CN VIII), and the anterior inferior cerebellar artery (AICA) were visualized (Figure 10D–F).

**Figure 11:**
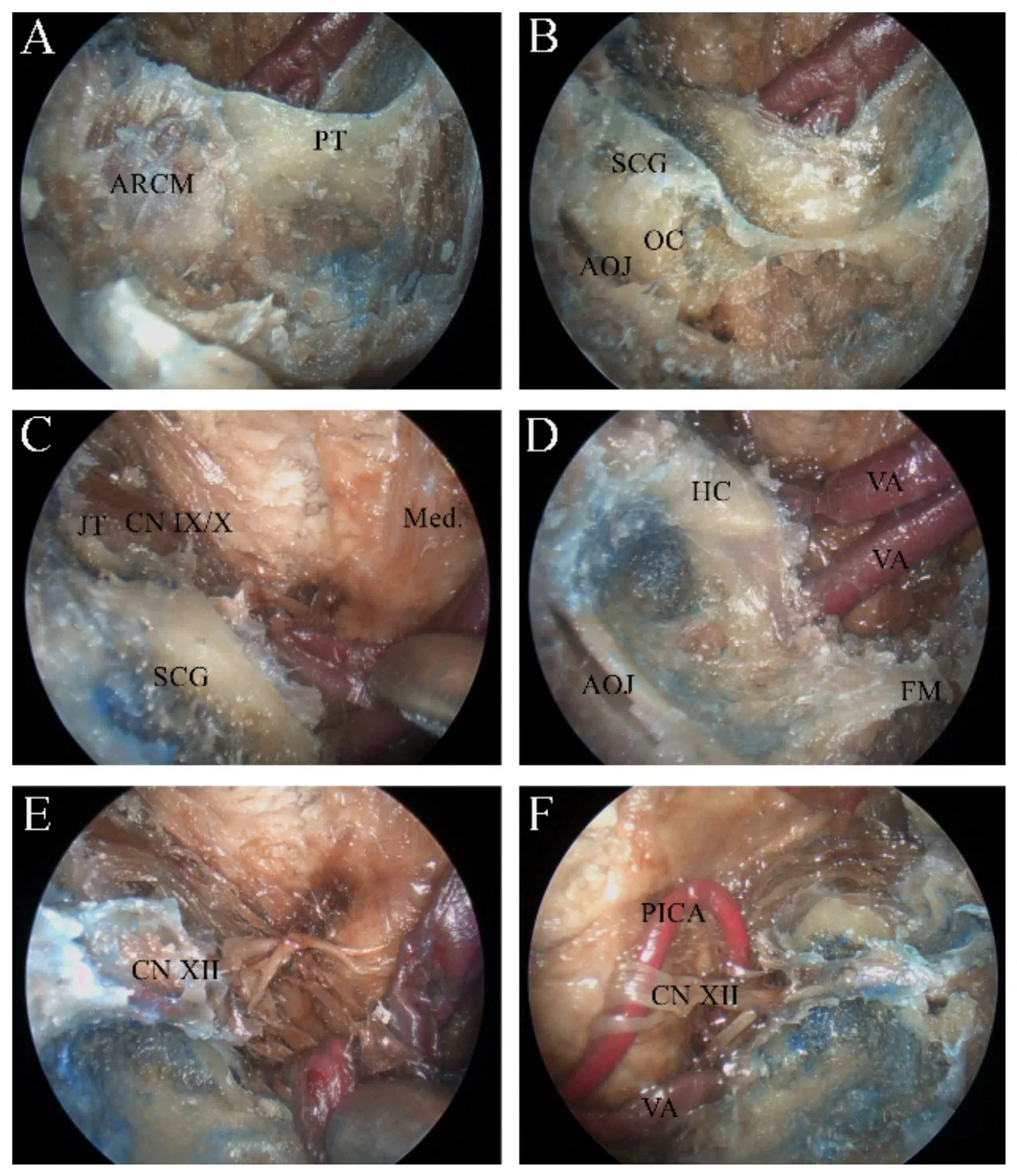
Stepwise dissection of the Inferior clival approach. (**A**) After removal of the PBF and the right LCM, the anterior rectus capitis muscle (ARCM) was exposed, which was observed covering the anterior aspect of the atlanto-occipital junction (AOJ) and attaching to the supracondylar groove (SCG) superior to the occipital condyle (OC). (**B**) The right ARCM was resected, exposing the AOJ, OC, and SCG. (**C**) Opening the dura over the inferior clivus revealed the greater part of the ventral medulla oblongata and the origin of the right lower cranial nerves. (**D**) Drilling posteriorly along the right SCG exposed the underlying hypoglossal canal (HC). (**E**,**F**) After drilling the anterior cortical bone of the HC, the hypoglossal nerve, the associated vascular structures, and the posterior inferior cerebellar artery (PICA) were exposed. AOJ, atlanto-occipital junction; ARCM, anterior rectus capitis muscle; CN IX, glossopharyngeal nerve; CN X, vagus nerve; CN XII, hypoglossal nerve; FM, foramen magnum; HC, hypoglossal canal; JT, jugular tubercle; Med., medulla oblongata; OC, occipital condyle; PICA, posterior inferior cerebellar artery; PT, pharyngeal tubercle; SCG, supracondylar groove; VA, vertebral artery.

Inferior clival/transcondylar approach: bounded laterally by the torus tubarius (TT) of the Eustachian tube (ET). The anterior rectus capitis muscle (ARCM) was resected, exposing the atlanto-occipital junction (AOJ), the occipital condyle (OC), and the supracondylar groove (SCG) (Figure 11A,B). The inferior clival bone was drilled away, and after dural opening, the medulla oblongata and the lower cranial nerves (CN IX-CN XII) were visualized (Figure 11C). Drilling posteriorly along the SCG exposed the hypoglossal canal (HC) and its internal contents (Figure 11D,E), and also revealed the posterior inferior cerebellar artery (PICA) (Figure 11F).

### 3.5. Craniocervical Junction and Odontoid Anatomy

**Figure 12:**
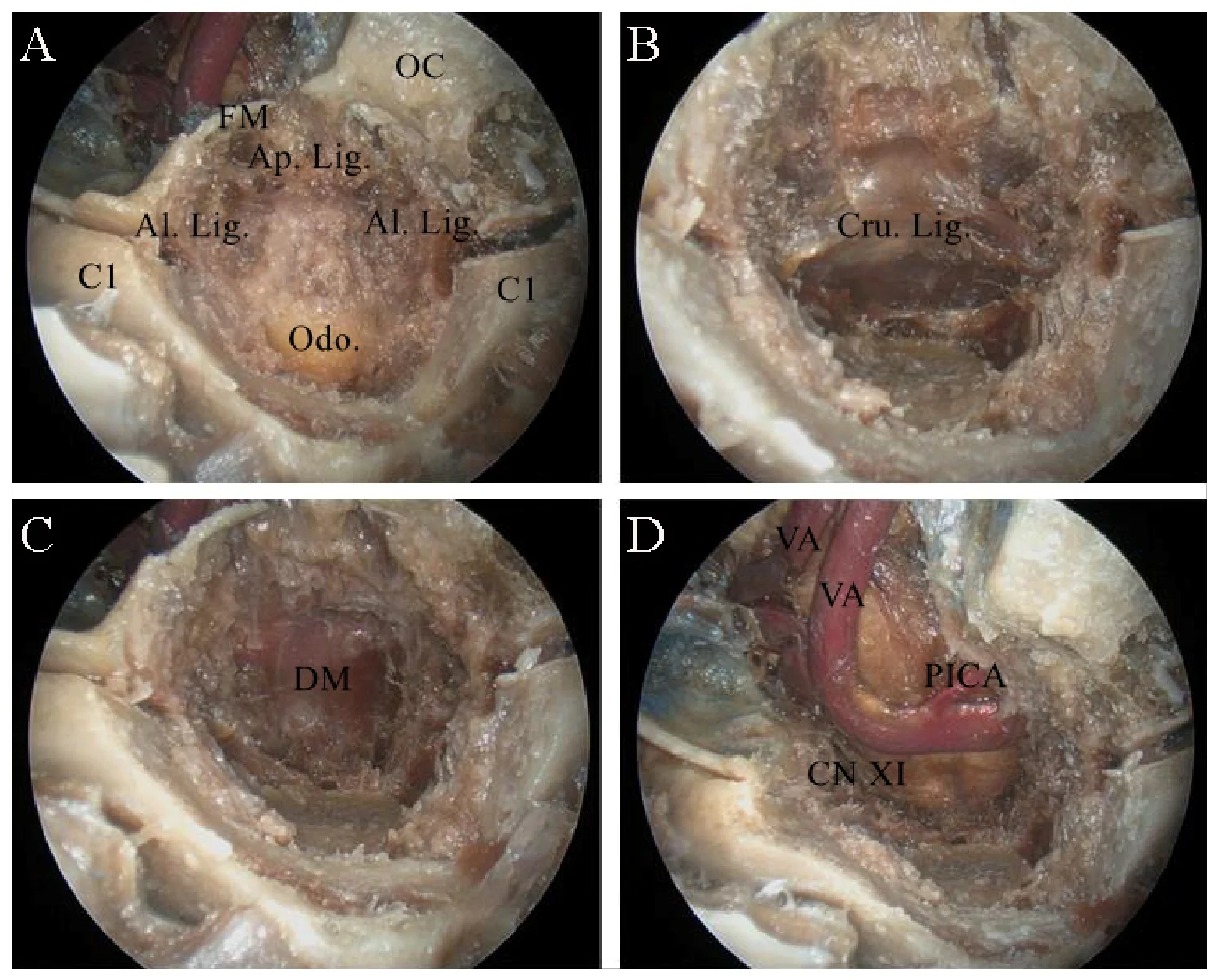
Stepwise dissection of the craniocervical junction. (**A**) Extensive fibrous tissues between the lower margin of the foramen magnum and the anterior arch of C1 were removed, exposing the bony and ligamentous structures of the Craniocervical Junction (CVJ). (**B**) The odontoid process was drilled away, revealing the underlying cruciate ligament. (**C**) The cruciate ligament and the overlying tectorial membrane were divided, exposing the underlying dura mater. (**D**) Following dural opening, the intradural structures were visualized. Al. Lig., alar ligament; Ap. Lig., apical ligament; C_1_, anterior arch of the atlas; CN XI, accessory nerve; Cru. Lig., cruciate ligament; DM, dura mater; FM, foramen magnum; OC, occipital condyle; Odo., odontoid process; PICA, posterior inferior cerebellar artery; VA, vertebral artery.

After removal of the anterior atlanto-occipital membrane and other fibrous tissues, the anterior arch of C1, the odontoid process, and the cruciate ligament were exposed (Figure 12A,B). Following odontoidectomy and dural opening, the vertebral artery (VA) and the accessory nerve (CN XI) were visualized (Figure 12C,D).

### 3.6. Anatomy of the Lateral Skull Base (Pterygopalatine Fossa, Jugular Foramen, and Infratemporal Fossa)

**Figure 13:**
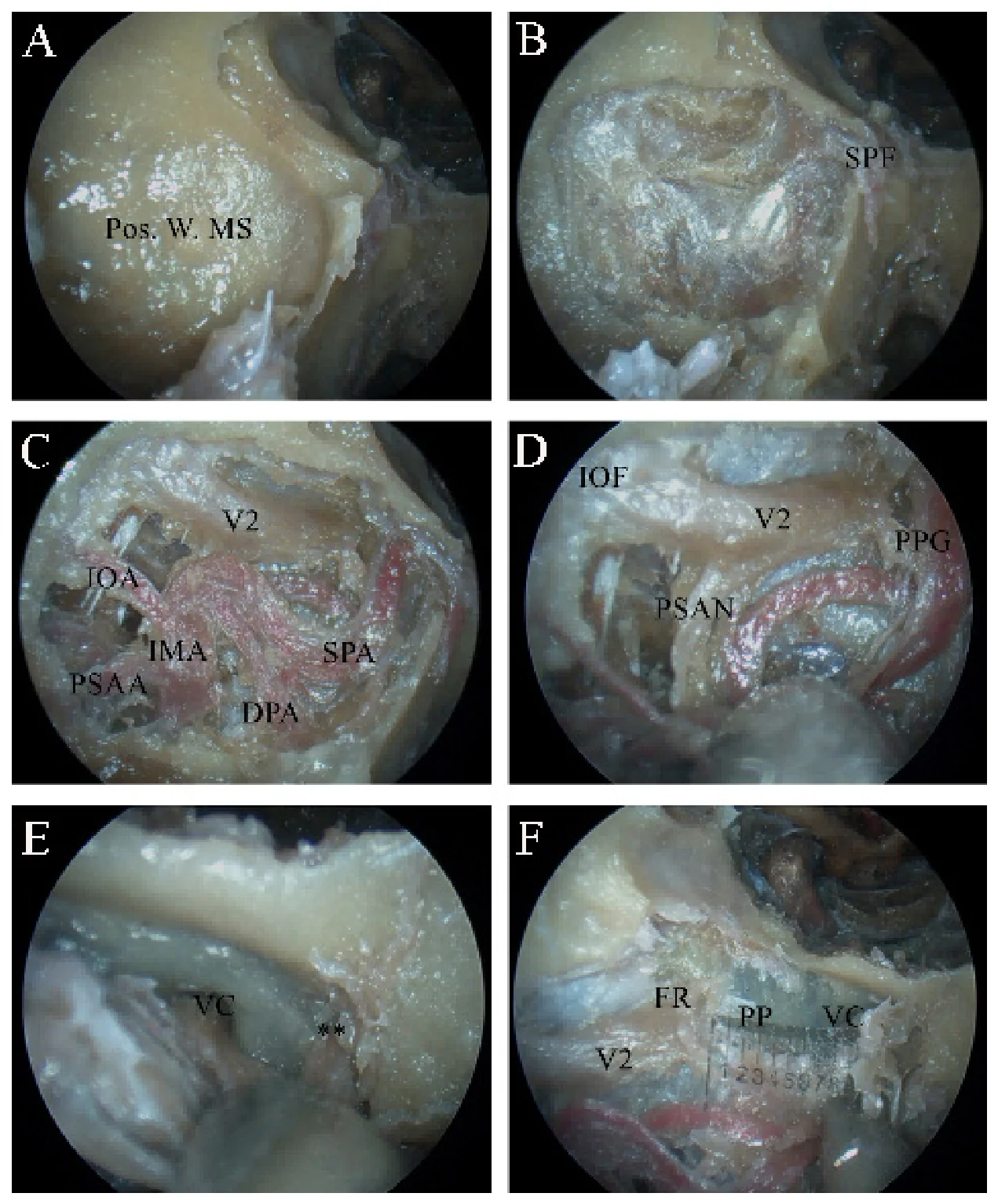
Stepwise dissection of the right pterygopalatine fossa. (**A**) The medial wall of the maxillary sinus was opened to enter the sinus cavity, exposing the posterior wall of the maxillary sinus. (**B**) The bone of the posterior maxillary wall was drilled away, revealing the sphenopalatine foramen (SPF) and the fascia covering the pterygopalatine fossa (PPF). (**C**) After removal of the fascia, the first-layer vascular structures within the PPF were exposed, demonstrating the third segment of the internal maxillary artery (IMA) and its branches. (**D**) The anterior vascular structures were retracted to expose the posterior neural elements, including the maxillary nerve (V2) and its branch, the posterior superior alveolar nerve (PSAN), as well as the pterygopalatine ganglion (PPG), coursing toward the inferior orbital fissure (IOF). (**E**) The vidian canal (VC) and the medial palatovaginal canal were identified at the roof of the PPF. (**F**) Further dissection exposed the foramen rotundum (FR), through which V2 exits the cranial cavity, along with the VC containing the vidian nerve. The region between the FR and the VC corresponds to the root of the pterygoid process. DPA, descending palatine artery; FR, foramen rotundum; IMA, internal maxillary artery; IOA, infraorbital artery; IOF, inferior orbital fissure; Pos. W. MS, posterior wall of the maxillary sinus; PP, pterygoid process; PPG, pterygopalatine ganglion; PSAA, posterior superior alveolar artery; PSAN, posterior superior alveolar nerve; SPA, sphenopalatine artery; SPF, sphenopalatine foramen; V2, maxillary division of the trigeminal nerve; VC, vidian canal; ** palatovaginal canal.

Pterygopalatine fossa (PPF): The posterior wall of the maxillary sinus and the sphenopalatine foramen (SPF) were opened, exposing the terminal branches of the internal maxillary artery (IMA) and the sphenopalatine artery (SPA) (Figure 13A–C). After removal of the adipose tissue, the vidian canal (VC), the vidian nerve (VN), and the maxillary division of the trigeminal nerve (V2) passing through the foramen rotundum (FR) into the cranial cavity were visualized (Figure 13D–F).

**Figure 14:**
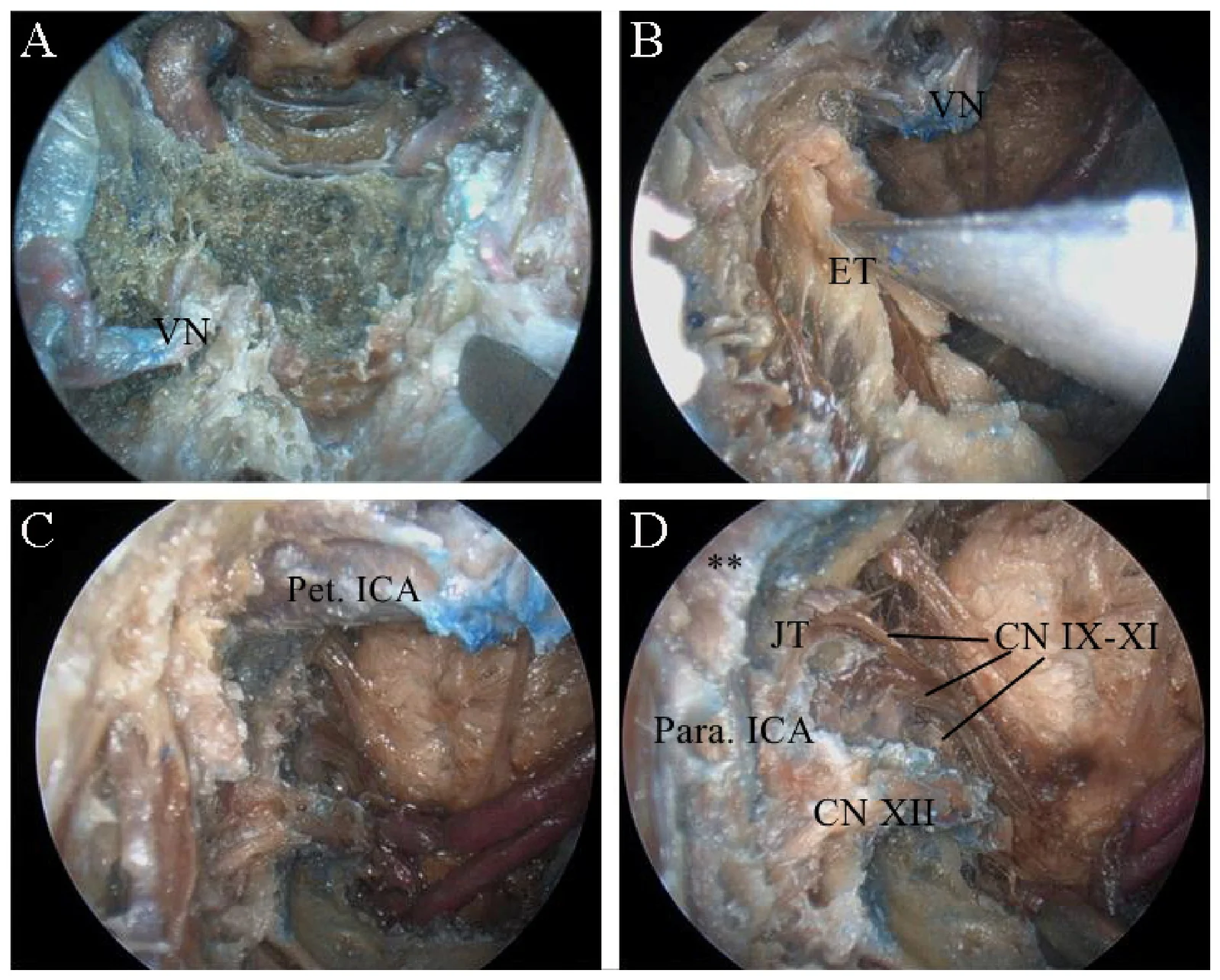
Complete exposure of the right jugular foramen. (**A**) Bone around the VC was drilled until the VN was completely mobilized. (**B**) The ET was completely transected from its attachment to the cartilaginous portion, exposing the more lateral aspect of the jugular tubercle (JT). (**C**) After transection of the ET, the intact horizontal petrous segment of the ICA was visualized. (**D**) Further drilling of the lateral portion of the JT exposed more of the cisternal segments of CN IX-CN XI, and also revealed the transition between the horizontal petrous segment and the parapharyngeal segment of the ICA. CN IX, glossopharyngeal nerve; CN X, vagus nerve; CN XI, accessory nerve; CN XII, hypoglossal nerve; ET, Eustachian tube; JT, jugular tubercle; Para. ICA, parapharyngeal segment of the internal carotid artery; Pet. ICA, horizontal petrous segment of the internal carotid artery; VN, vidian nerve; ** transition between the horizontal petrous ICA and the parapharyngeal ICA.

Jugular foramen (JF): Bone around the VC was drilled to mobilize the VN (Figure 14A). The cartilaginous portion of the Eustachian tube (ET) was transected (Figure 14B), exposing the horizontal petrous segment of the ICA (Figure 14C). The lateral aspect of the jugular tubercle (JT) was drilled away, revealing the cisternal segments of the lower cranial nerves (CN IX-CN XI) (Figure 14D).

**Figure 15:**
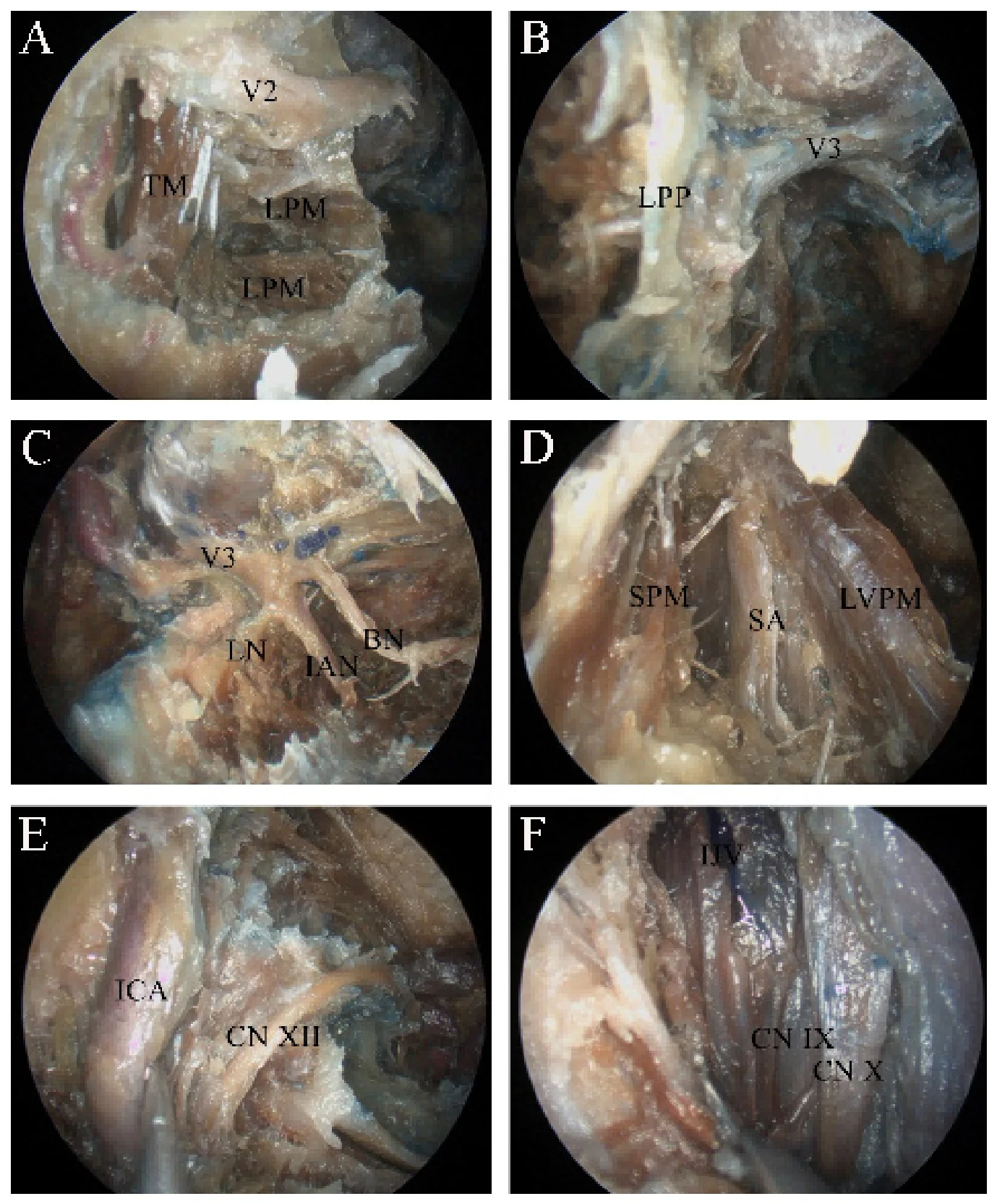
Anatomy of the infratemporal fossa and the upper parapharyngeal space. (**A**,**B**). After removal of the neurovascular structures within the right PPS, the masticator space was exposed posteriorly, revealing the upper and lower heads of the lateral pterygoid muscle (LPM) attaching to the lateral pterygoid plate (LPP), with the temporalis muscle located laterally. (**C**) Left side: the LPM was transected to expose V3 and its branches. (**D**) Right side: after transection of the ET, the underlying levator veli palatini muscle (LVPM), the stylopharyngeal fascia, and the stylopharyngeus muscle (SPM) were exposed medially. (**E**) After transection of the right LVPM, the stylopharyngeal fascia was opened from medial to lateral, exposing the parapharyngeal segment of the ICA and the hypoglossal nerve posteriorly. (**F**) The right retrostyloid space was exposed, revealing the internal jugular vein (IJV), CN IX and CN X. BN, buccal nerve; CN IX, glossopharyngeal nerve; CN X, vagus nerve; CN XII, hypoglossal nerve; ET, Eustachian tube; IAN, inferior alveolar nerve; ICA, parapharyngeal segment of the internal carotid artery; IJV, internal jugular vein; LN, lingual nerve; LPM, lateral pterygoid muscle; LPP, lateral pterygoid plate; LVPM, levator veli palatini muscle; SA, stylopharyngeal fascia; SPM, stylopharyngeus muscle; TM, temporalis muscle; V2, maxillary division of the trigeminal nerve; V3, mandibular division of the trigeminal nerve.

Infratemporal fossa (ITF) and upper parapharyngeal space: The lateral pterygoid muscle (LPM) and the lateral pterygoid plate (LPP) were removed, exposing the mandibular division of the trigeminal nerve (V3) and its branches (Figure 15A–C). After opening the stylopharyngeal fascia (SA), the parapharyngeal segment of the ICA, the internal jugular vein (IJV), and CN IX, X, and XII were visualized (Figure 15D–F).

## 4. Results

In specimens with poor sphenoid sinus pneumatization, the classic anatomical landmarks within the sphenoid sinus cavity—including the sellar floor, optic nerve prominence, ICA prominence, and the clival recess—were only partially identifiable, and their prominences were indistinct (Figure 1D). The optic nerve prominence could not be identified in any of the poorly pneumatized specimens. Therefore, morphometric measurements of constant osseous structures were performed.

### 4.1. Identification Rate and Constant Interosseous Distance of the VC-FR Complex

Among the 20 poorly pneumatized sides, the VC and its contiguous VN were endoscopically identifiable in all 20 sides (20/20, 100%). In contrast, the ICA prominence and the optic nerve prominence were completely identifiable in 0 sides (0/20, 0%). The VC-FR complex was used as the primary osseous landmark for subsequent morphometric measurements. Quantitative measurements revealed that the length of the VC was 17.1 ± 1.9 mm on the left and 17.8 ± 2.1 mm on the right (*p* = 0.455), and the distance from the VC to the FR was 7.8 ± 0.9 mm on the left and 8.0 ± 1.0 mm on the right (*p* = 0.710), with no statistically significant bilateral differences. The standard deviations of these distances were all less than 1.0 mm (Table 1), indicating a highly constant anatomical position with minimal variability.

### 4.2. Morphometric Analysis of the Clivus and Midline Structures

Overall clivus: The distance from the sellar floor to the basion was 27.6 ± 1.2 mm, and the total clival length was 28.7 ± 2.1 mm. The widths of the superior, middle, and inferior clivus were 20.8 ± 2.0 mm, 22.7 ± 2.8 mm, and 29.2 ± 3.9 mm, respectively, showing a gradual widening from superior to inferior. Foramen magnum (FM) and pharyngeal tubercle: The transverse diameter of the FM was 30.5 ± 2.2 mm, and the distance from the pharyngeal tubercle (PT) to the basion was 12.4 ± 2.6 mm (Table 2). Resectable areas: The total resectable area of the entire clivus was 8.6 ± 1.1 cm^2^; the resectable area of the dorsum sellae was 1.29 ± 0.44 cm^2^; and the sum of the two was 9.76 ± 1.01 cm^2^ (Table 3).

### 4.3. Safe Zone of the OC and HC

As shown in Table 4, the mean length of the left OC was 24.7 ± 1.9 mm versus 23.8 ± 2.6 mm on the right (*p* = 0.363); the width was 13.4 ± 2.0 mm on the left and 13.9 ± 2.3 mm on the right (*p* = 0.550); and the thickness was 10.7 ± 1.9 mm versus 10.4 ± 2.1 mm, respectively (*p* = 0.746). No statistically significant bilateral differences were observed; however, given the limited sample size, this should not be interpreted as proof of symmetry. The distance from the HC to the jugular tubercle (JT) was 7.8 ± 1.5 mm on the left and 7.3 ± 1.1 mm on the right (*p* = 0.447); the distance from the HC to the basion was 18.5 ± 1.8 mm versus 18.2 ± 1.4 mm, respectively (*p* = 0.711).

### 4.4. Quantitative Assessment of Safe Drilling of the PA

The total volume of the left PA was 2.27 ± 0.31 cm^3^ versus 2.05 ± 0.20 cm^3^ on the right (*p* = 0.070). The resectable PA volume via the expanded EEA was 1.01 ± 0.12 cm^3^ on the left and 0.99 ± 0.13 cm^3^ on the right (*p* = 0.600), with an average resection proportion ranging from 45.3 ± 8.0% to 48.2% ± 4.0% (Table 5).

## 5. Discussion

This study systematically addresses the paucity of quantitative anatomical data for the expanded EEA in the context of poor skull base pneumatization. Our principal finding is that when the sphenoid sinus and clivus are poorly pneumatized, the mucosal- and thin bone-dependent “prominence” landmarks—including the ICA prominence, optic nerve prominence, and clival recess—are completely unreliable. Consequently, a paradigm shift to a “coordinate-based navigation” strategy, grounded in deeper osseous structures (VC, FR, HC, and SCG), is essential.

### 5.1. Poor Skull Base Pneumatization: Clinical Challenges and Current Research

Pneumatization of the sphenoid sinus is a progressive process with considerable individual variability in its extent. According to the classification proposed by Hammer and Radberg, sphenoid sinus pneumatization can be categorized into four types: conchal, presellar, sellar, and postsellar. Among these, the conchal and presellar types fall within the spectrum of poor pneumatization, and their respective incidences reported in the literature vary slightly [6,7,8,14]. Vaezi et al. [15] proposed three types of sphenoid sinus pneumatization based on the degree of pneumatization on coronal images in relation to the VC and the FR: Type I—pneumatization extending from the midline to the medial border of the VC (accounting for 25%); Type II—pneumatization extending to the medial border of the FR (39%); and Type III—pneumatization extending beyond the medial border of the FR (37%). This classification enables prediction of the distance between the VC and the CN V2, thereby guiding the size of the surgical window for transnasal access to the middle cranial fossa. Kiciński et al. [16] analyzed sphenoid sinus pneumatization in children under 18 years of age and classified it into three types according to the extent of sinus extension into the various processes of the sphenoid bone: pneumatization of the lesser wing was observed in 19.44% of cases, of the greater wing in 36.11%, and of the pterygoid process in 25.00%.

The impact of poor pneumatization on EEA has garnered considerable clinical attention. Kang et al. [9], based on CT findings from 490 patients, reported that when sphenoid sinus pneumatization is poor, the ICA prominence is indistinct, and the intersphenoid septum (ISS) is often attached to or closely adjacent to the paraclival ICA. This anatomical variation implies the loss of the ICA prominence as a warning signal during surgery, thereby significantly increasing the risk of ICA injury. Kosugi et al. [10] further demonstrated that the degree of pneumatization of the LRSS directly affects the resection rate of tumors in the cavernous sinus region, and thus preoperative assessment of LRSS development is crucial for surgical planning. Given the unreliability of the ICA prominence, the identification of a constant surrogate landmark becomes paramount. The VC/VN complex fulfills this role.

### 5.2. The VC/VN: A Critical Landmark in the Absence of Adequate Pneumatization

Given that the loss of the ICA prominence precludes the use of the carotid artery itself as a landmark, we sought to identify the most constant surrogate osseous landmarks that maintain a fixed spatial relationship with the ICA. The VC and FR were selected for this purpose, as they are located at a consistent distance from the petrous ICA and are unaffected by the degree of pneumatization. In well-pneumatized sphenoid sinuses, the ICA prominence is distinctly visible; however, in cases of poor pneumatization, this prominence is frequently absent. Our measurements confirm that the VC is highly constant in its anatomy (approximately 17 mm in length), and its fixed distance from the FR (approximately 8 mm) constitutes a rectangular coordinate axis. These small standard deviations (all <1.0 mm) suggest that the VC-FR complex maintains a highly constant anatomical position, which supports its use as a reliable landmark. The data support that the VC-FR complex represents the most reliable rigid osseous landmark for localization under conditions of poor pneumatization. Dissection along the medial and inferior aspect of the VN may assist in approximating the position of the horizontal petrous segment of the ICA and the FL [17].

### 5.3. The Four-Compartment Theory of the CS and the Endoscopic “Triangle Safe Zones”

We validated the CS compartments from the EEA proposed by Fernandez-Miranda et al. [3]. In the setting of poor pneumatization, the CN III, IV, and V1 within the lateral wall of the CS become more difficult to identify due to the lack of an osseous backdrop. Through compartmental dissection, we found that the anteromedial triangle of the CS and the quadrangular space represent the only reliable corridors for accessing the middle cranial fossa under poorly pneumatized conditions [18,19]. Intraoperatively, one should preferentially search for the sympathetic plexus inferior to the horizontal segment of the ICA (i.e., in the inferior compartment), or locate the CN VI, which lies at the most inferior aspect of the posterior compartment, behind the ICA, thereby indirectly confirming the ICA position in a reverse manner.

### 5.4. Petrous Apex Drilling and the Abducens Nerve

The PA is an obligatory corridor for accessing lesions with lateral extension in the middle clival region. In the present series, the mean resectable PA volume via the expanded endoscopic endonasal approach was approximately 1.0 cm^3^, corresponding to 45–48% of the total PA volume. However, in one specimen, the resected volume reached 63.8%, indicating that the extent of drilling varied considerably among individuals.

Anatomically, the abducens nerve (CN VI) courses within Dorello’s canal, immediately medial to the PA and inferior to Gruber’s ligament [20]. This segment of the nerve has extremely limited mobility, and it is therefore the structure most vulnerable to injury during drilling of the PA. Because this was a cadaveric study, we did not assess whether drilling beyond a certain proportion caused neurovascular injury; consequently, no validated safety threshold can be proposed. Nevertheless, based on the anatomical relationship observed, the superior margin of the CN V_2_ branches may serve as a useful landmark to define the upper limit of drilling, and careful identification of the CN VI is essential to avoid direct mechanical or thermal injury.

These observations should be interpreted as descriptive anatomical data rather than a prescriptive surgical rule. Clinical studies with intraoperative neuromonitoring and postoperative outcome assessment are warranted to determine the safe extent of PA drilling in patients with poor pneumatization.

### 5.5. Occipital Condyle and Hypoglossal Canal: Anatomical Observations

The inferior clival and CVJ region represents the deepest and most structurally complex area in this anatomical territory. We support the viewpoint of Morera et al. [13] and further provide bilateral paired data that did not show statistically significant bilateral differences between the OC and HC. The supracondylar groove (SCG) was consistently identified as a reliable surface landmark for locating the HC. In our dissections, the HC orifice was located immediately posterior to the SCG. However, the exact distance from the articular surface of the OC to the SCG was not systematically measured in this study; therefore, no numerical rule or safety threshold can be proposed. Nevertheless, the consistent anatomical relationship observed suggests that the SCG may serve as a useful intraoperative guide for approximating the position of the HC during transcondylar drilling. These observations should be regarded as descriptive anatomical data. Future studies with systematic measurement of the OC-SCG distance and clinical correlation are warranted to determine the safe extent of condylar drilling and to preserve the hypoglossal nerve and craniocervical stability [21].

### 5.6. Approach-Related Limitations of the Lateral Skull Base (JF and ITF)

For lesions involving the JF and ITF, the ET serves as a critical landmark. Transection of the ET allows adequate exposure of the transition zone between the horizontal petrous segment and the parapharyngeal segment of the ICA [22]. However, it should be noted that our anatomical findings indicate this is a very narrow and long corridor (approximately 8–10 cm). The loss of osseous landmarks due to poor pneumatization may increase the difficulty of hemostasis and the risk of postoperative swallowing dysfunction; however, this inference is based on anatomical observations rather than clinical outcomes, and further clinical studies are required to confirm these potential risks. Therefore, for patients with poor pneumatization in this region, rigorous preoperative evaluation of the vascular course (CTA) is recommended, and preparation for conversion to an open approach should be made.

### 5.7. Limitations of This Study

Several limitations of this study should be acknowledged. First, the sample size was relatively small (10 cadaveric heads, 20 sides), which may limit statistical power and the generalizability of the findings. Second, preoperative imaging data (such as CT or MRI) were not available; therefore, the definition of poor pneumatization was based on surgical observation rather than radiological classification, and the degree of pneumatization could not be objectively quantified. Third, because all specimens in this study were classified as poorly pneumatized, no well-pneumatized control group was available. Therefore, the comparison of landmark visibility between poorly and well-pneumatized conditions was based on descriptive observation rather than statistical testing. Future studies with both types of specimens are warranted to validate these findings. Fourth, all measurements were performed by a single operator using conventional tools (flexible ruler and vernier caliper). Areas and volumes were estimated from linear dimensions using geometric formulas rather than direct three-dimensional volumetry. This simplified approach may introduce measurement error, particularly for irregular bony cavities. In addition, the relationship between the extent of PA drilling and neurovascular injury was not assessed; the exact distance from the OC articular surface to the SCG was not systematically measured; therefore, no numerical rule or safety threshold can be proposed. Finally, clinical outcomes such as postoperative cerebrospinal fluid leakage and neurological deficits were not assessed; therefore, the direct impact of these anatomical findings on clinical outcomes remains to be established.

## 6. Conclusions

Poor skull base pneumatization may increase the difficulty of intraoperative localization and the risk of neurovascular injury during endoscopic endonasal surgery. Through systematic cadaveric dissections and quantitative analyses, the present study suggests that, in the absence of pneumatized air-cell bulges as landmarks, it is essential to rely on the VC-FR complex for ICA localization, to use the SCG-HC relationship for safe drilling of the inferior clivus, and to strictly limit the PA drilling volume to approximately 1.0 cm^3^ to protect the CN VI. These morphometric data, based on absolute osseous coordinates, provide a solid anatomical baseline for the precise and safe performance of expanded EEA for various skull base tumors in the setting of poor pneumatization.

## Figures and Tables

**Table 1:** Morphometric analysis of VC, FR and OCR (mm).

Parameter	Max		Min		Mean		SD		*p*-Value
	L	R	L	R	L	R	L	R	
VC length	20.6	21.1	14.8	15.0	17.1	17.8	1.9	2.1	0.455
Distance between VC and FR	9.2	9.4	6.4	6.8	7.8	8.0	0.9	1.0	0.710
Distance between bilateral VCs	22.3		18.0		20.2		1.8		
Distance between medial OCRs	13.2		9.1		10.9		1.5		

FR, foramen rotundum; OCR, opticocarotid recess; VC, vidian canal; L, Left; R, Right; SD, standard deviation.

**Table 2:** Morphometric Assessment of the Central Clival Area (mm).

Parameter	Max	Min	Mean	SD
Distance from sellar floor to basion	29.2	26.1	27.6	1.2
Clival length	32.4	25.8	28.7	2.1
Superior clival width	25.3	17.7	20.8	2.0
Middle clival width	26.3	18.9	22.7	2.8
Inferior clival width	36.4	24.5	29.2	3.9
Distance between bilateral OC (Sup.)	23.5	16.2	20.3	2.5
Distance between bilateral OC (Mid.)	26.3	18.6	22.4	2.7
Distance between bilateral OC (Inf.)	47.8	38.6	43.2	3.4
Intracranial distance between bilateral HC	30.2	24.5	26.4	1.8
Extracranial distance between bilateral HC	36.3	31.3	34.2	1.6
Transverse diameter of FM	34.2	28.7	30.5	2.2
Distance from PT to basion	18.8	9.8	12.4	2.6

The vertical span of the clivus was measured from the sellar floor (superior limit) to the basion (inferior limit). Superior clival width was measured at the level of the sellar floor; middle clival width at the level of the pharyngeal tubercle; inferior clival width at the level of the anterior margin of the foramen magnum. Bilateral boundaries were defined by the medial margins of the paraclival ICA (superior and middle clivus) and the medial margins of the OC (inferior clivus). FM, foramen magnum; HC, hypoglossal canal; OC, occipital condyle; PT, pharyngeal tubercle; Sup., superior; Mid., middle; Inf., inferior.

**Table 3:** Resectable areas of the clivus and dorsum sellae (cm^2^).

Parameter	Max	Min	Mean	SD
Resectable area of the clivus	10.4	6.8	8.6	1.1
Resectable area of the dorsum sella	2.22	0.79	1.29	0.44
Total resectable area of both	11.56	7.95	9.76	1.01

Total resectable area was calculated as the sum of the clival and dorsum sellae areas for each individual specimen; summary statistics were then derived from these per-specimen totals. Therefore, the total values do not equal the arithmetic sum of the corresponding summary statistics in the rows above.

**Table 4:** Morphometric Analysis of the OC and HC (mm).

Parameter	Max		Min		Mean		SD		*p*-Value
	L	R	L	R	L	R	L	R	
OC length	28.4	27.2	22.6	20.5	24.7	23.8	1.9	2.6	0.363
OC width	16.8	18.9	10.0	10.0	13.4	13.9	2.0	2.3	0.587
OC thickness	13.2	14	8.0	7.8	10.7	10.4	1.9	2.1	0.746
Distance from HC to JT	10.2	9.4	5.8	5.6	7.8	7.3	1.5	1.1	0.447
Distance from HC to basion	21.2	20.8	15.9	16.2	18.5	18.2	1.8	1.4	0.711

HC, hypoglossal canal; JT, jugular tubercle; OC, occipital condyle; L, Left; R, Right; SD, standard deviation.

**Table 5:** Quantitative analysis of endoscopic PA drilling.

Parameter	Max		Min		Mean		SD		*p*-Value
	L	R	L	R	L	R	L	R	
Total PA volume (cm^3^)	2.68	2.35	1.82	1.78	2.27	2.05	0.31	0.20	0.070
Resected volume (cm^3^)	1.25	1.16	0.83	0.78	1.01	0.99	0.12	0.13	0.600
Resection percentage (%)	63.8	52.7	37.7	38.6	45.3	48.2	8.0	4.0	0.331

PA, petrous apex.

## Data Availability

The original contributions presented in this study are included in the article. Further inquiries can be directed to the corresponding authors.

## Abbreviations

ACA	Anterior cerebral artery
AOJ	Atlanto-occipital junction
ARCM	Anterior rectus capitis muscle
BA	Basilar artery
CR	Clival recess
CS	Cavernous sinus
CVJ	Craniocervical junction
EEA	Endonasal endoscopic approach
ET	Eustachian tube
FL	Foramen lacerum
FM	Foramen magnum
FR	Foramen rotundum
HC	Hypoglossal canal
ICA	Internal carotid artery
IJV	Internal jugular vein
IMA	Internal maxillary artery
IT	Inferior turbinate
ITF	Infratemporal fossa
JF	Jugular foramen
JT	Jugular tubercle
LCM	Longus capitis muscle
LPM	Lateral pterygoid muscle
LPP	Lateral pterygoid plate
MB	Mammillary body
MT	Middle turbinate
OC	Occipital condyle
OCR	Opticcarotid recess
ON	Optic nerve
PA	Petrous apex
PBF	Pharyngobasilar fascia
PICA	Posterior inferior cerebellar artery
PPF	Pterygopalatine fossa
PS	Planum sphenoidale
PT	Pharyngeal tubercle
SCG	Supracondylar groove
SO	Sphenoid ostium
SPA	Sphenopalatine artery
SPF	Sphenopalatine foramen
ST	Superior turbinate
TS	Tuberculum sellae
VA	Vertebral artery
VC	Vidian canal
VN	Vidian nerve
